# Challenges faced by nurses in using pain assessment scale in patients unable to communicate: a qualitative study

**DOI:** 10.1186/s12912-018-0281-3

**Published:** 2018-03-16

**Authors:** Kolsoum Deldar, Razieh Froutan, Abbas Ebadi

**Affiliations:** 1 Department of Medical Informatics, Faculty of Medicine, Mashhad University of Medical Sciences, Mashhad, Iran; 2 Department of Medical-Surgical Nursing, School of Nursing and Midwifery, Mashhad University of Medical Sciences, Mashhad, Iran; 3 Behavioral Sciences Research Center, Faculty of Nursing, Baqiyatallah University of Medical Sciences, Tehran, Iran

## Abstract

**Background:**

One helpful strategy adopted for pain management in non-verbal, intubated patients is the use of a proper pain assessment scale. The purpose of the present study is to achieve a better and deeper understanding of the existing nurses’ challenges in using pain assessment scales among patients unable to communicate.

**Methods:**

This qualitative study was conducted using content analysis. Purposive sampling was used to select the participants and continued until data saturation. The participants included 20 nurses working in intensive care units. Data was collected using semi-structured interviews and analysis was done using an inductive approach.

**Results:**

Four categories and ten sub-categories were extracted from the experiences of the nurses working in the intensive care units in terms of nursing challenges in using non-verbal pain assessment scales. The four categories included “forgotten priority”, “organizational barriers”, “attitudinal barriers”, and “barriers to knowledge”.

**Conclusions:**

The findings of the present study have shown that various factors might influence on the use of non-verbal pain assessment scales in patients unable to communicate. Identifying these challenges for nurses can help take effective steps such as empowering nurses in the use of non-verbal pain assessment scales, relieving pain, and improving the quality of care services.

## Background

Pain is an unpleasant sensory and emotional experience associated with actual or potential tissue damage or described in terms of such damage [1]. It is a common phenomenon and a major stressor in intubated patients [2–4]. Various reasons other than the original disease, e.g. endotracheal tube suctioning, chest tube insertion, respiratory exercises, coughs, and certain positions on the bed, can cause pain [5–7]. Despite advances in theories related to pain control [8–11], pain is still a major problem in critically ill patients admitted to intensive care units (ICU) and 40–77.4% of ICU patients complain about the experience of pain [12, 13]. Since these patients may suffer from numerous neurological, physiological, and communicative disabilities arising from a variety of reasons including dependence on a mechanical ventilator (MV) and concurrent use of sedatives, they may not be able to accurately estimate the level of their pain [14, 15]. Inappropriate diagnosis of pain experienced by ICU patients is also associated with complications such as increased risk of infection, prolonged MV, hemodynamic disorders, paranoia, immune-suppression, and even death [16–18].

Some researchers believe that the most reliable method of pain evaluation is the patient’s self-report [16]. But if patient doesn’t have enough ability to provide verbal self-report of pain (e.g. ICU patients), it is recommended to use other available methods for pain management [14].

The first step in the management of pain is its diagnosis and evaluation [19], i.e. a reliable pain assessment tool is essential to efficient pain management [14, 20–22]. Such a tool can contribute to correct decision-making during pain management [23, 24] and promote pain diagnosis and evaluation [25]. Therefore, an effective pain assessment scale should be a part of the recording process system. Since evaluation is a basic principle in nursing care and it can form the foundation for nursing interventions, each hospital should have a practical approach to pain measurement [26]. A variety of pain measurement tools, including the Visual Analogue Scale (VAS), Numeric Rating Scale (NRS), Verbal Descriptor Scale (VDS), Smiling Face Scale (SFS), and Numeric Descriptor Scale (NDS), can be used to determine the severity of pain and its related behaviors [27–30]. In addition, the Behavioral Pain Scale (BPS), Critical-Care Pain Observation Tool (CPOT), and Nonverbal Pain Scale (NVPS) can be administered to screen pain in critically ill ICU patients who are unable to communicate [31, 32]. This group of patients may include unconscious, sedated, or intubated patients, as well as those with reduced consciousness levels, communication barriers, or head trauma [10, 33]. However, there are few documents on the use of such scales. According to G’elinas et al. (2004), pain assessment scales were only employed in 1.6% of the 183 events recorded for intubated patients. Although evaluation of pain behaviors was common (reported in 73% of cases), such evaluations and observations were conducted without any valid and reliable tools [34]. In a study on 3601 critically ill intubated patients, Payen et al. (2007) found that pain was not assessed in 53% of the patients who had received pain-killers. Moreover, only 28% of pain evaluations were performed through appropriate and specific pain assessment tools [35].

Since all patients under MV receive analgesics or sedatives, mostly narcotic drugs, pain assessment scales for these patients have not received adequate attention [36]. It seems that efficient pain evaluation and management for critically ill patients has become a major challenge for ICU nurses [21]. Therefore, considering the role of nurses as the main individuals involved in pain evaluation and management, this study sought to address the nurses’ challenges in the use of pain assessment tools in patients unable to communicate.

## Methods

### Study design

This qualitative study was conducted using content analysis. The researchers performed an in-depth direct analysis of experiences of ICU nurses. The findings are presented as codes, subcategories, and categories using an inductive approach [37].

### Participants and study setting

The selection of participants was performed using a purposeful sampling method. 20 interviews were conducted with nurses working in ICUs. Subject selection was conducted with maximum variation in personal factors (age, education level, duration of work experience, and organizational role). Data was collected using semi-structured interviews, and analysis was done using an inductive approach. All study participants were interested in sharing their experiences.

### Ethical considerations

This study was approved by the Ethics Committee of Mashhad University of Medical Sciences in May 2016 (code: IR.MUMS.REC.1395.159). Moreover, the participants were ensured of data confidentiality and autonomy. They were informed of the purpose of the study and the voluntary nature of their participation. A written consent was obtained from all participants before recording the interviews.

### Data collection and analysis

Content analysis was performed on Persian transcripts, before translation. The interviews were started with a number of general questions (e.g. “Please describe one of your experiences of one day working in the ICU.”) and continued with more specific questions (e.g. “Please speak about your own experiences of pain management in patients unable to communicate.”, “Please describe your experiences of using non-verbal pain scales.”, and “What problems and issues do you face?”). Individual semi-structured interviews were conducted in a private room at the participants’ workplace.

Based on the Graneheim and Lundman’s method [37], the analysis process consisted of the following steps:

1. The recorded interviews were transcribed and read to get an overall understanding.
2. The texts were divided into meaningful units.
3. The meaningful units were extracted and encoded.
4. Based on their similarities and differences, the initial codes were classified into subcategories.

During the open coding stage, all the transcripts were reread closely and thoroughly for several times and the keywords, expressions, incidents, and actualities were noted. The basic codes were taken, and the codes and all extracted data were compared to identify the existing similarities and differences. Afterward, the categories and subcategories were created. A preparatory arrangement of codes, categories, and subcategories was framed from the first interview, and the developing codes were considered as the outcomes.

### Trustworthiness

Maximum variation sampling, member checking, and peer questioning and cross-examination were used to ensure the trustworthiness, dependability, and credibility of the data, respectively. In order for member checking, each participant was provided with the transcript of his/her coded interview along with a summary of the extracted themes and asked to determine whether the codes are representative of and matched with their experiences. Peer checking of the transcripts was conducted by two faculty members with a PhD in nursing. They received the transcripts and followed the above-mentioned process to reach the core themes. The obtained inter-rater agreement was equal to or above 90%.

The long presence of the authors in the field (from May 2016 to Apr 2017) enabled them to win the participants’ trust and develop strong communication links with the interviewees. This facilitated precise data collection.

## Results

The study sample consisted of 20 ICU nurses (nine men and 11 women). The mean age and mean work experience were 35.7 ± 6.1 and 12.3 ± 6.1 years, respectively. Other details are available in Table 1.

**Table 1:** Summary of participant characteristics

Variables	Status	Percent
Gender	Females	55%
	Males	45%
Educational Degree	Bachelor’s	90%
	Master’s or higher	10%

The factors inhibiting the use of pain assessment scales in patients unable to communicate were grouped into four categories including “forgotten priority”, “organizational barriers”, “attitudinal barriers”, and “barriers to knowledge” (Table 2).

**Table 2:** The main categories and related sub-categories

Category	Sub-category
Forgotten priority	Non-routine pain assessment/evaluation
	Inadequate physician-nurse interaction in terms of patient pain
	Absence of non-verbal pain assessment scales in the nursing flowchart
	Lack of policies and clinical guidelines
Organizational barriers	Inadequate nurse-patient ratio
	Presence of less experienced personnel
Attitudinal barriers	Adequacy of sedatives
	Failure to understand pain in unconscious patients
	No belief in non-verbal pain assessment scales
Barriers to knowledge	Unfamiliarity with the use of non-verbal pain assessment scales
	Insufficient training for clinical use of pain assessment scales

The findings along with their related quotes are shown below:

## Forgotten priority

One of the concepts extracted from data analysis based on the experiences of our participants was “forgotten priority”. This category consisted of four subcategories including: “non-routine pain assessment/evaluation”, “inadequate physician-nurse interaction regarding patient pain”, “absence of non-verbal pain assessment scales in the nursing flowchart”, and “lack of relevant policies and clinical guidelines”.

Due to non-routine pain assessment/evaluation in patients unable to communicate, nurses did not use pain measurement scales for these patients. As participant #8 stated:

*“… I have been working in the ICU for about 7 years… almost all the duties in our shifts are routine... care for the airway and attention to the alarms of the mechanical ventilators... during this time I have not performed evaluation of pain for patients with decreased level of consciousness (LOC)... Well, until now, pain evaluation and recording have not been conducted routinely for these patients… therefore, there has been no necessity to use non-verbal pain assessment scales...”*

The second category of “forgotten priority” was inadequate physician-nurse interaction regarding patient pain. Despite the fact that pain management is an important patient right and a health-care priority, patient pain is seldom mentioned during the visit time. Participant #13 mentioned that:

*“...during the visits of intubated patients experiencing decreased LOC; test results, respiratory mode, and so on are discussed and there are not talks about patient pain and its evaluation results… well, this situation can impact the use of non-verbal pain assessment scales for such patients...”*

Given the absence of non-verbal pain assessment tools in the nursing flowchart, the nurses believed that no place (in patient record or nursing flowchart) was specified for the use of these standardized tools despite the importance of pain relief in patients under MV. Participant #20 indicated that:

*“... We can record the results of arterial blood gases, blood tests, vital signs, and nursing reports in the nursing flowchart… however, no place has been specified for non-verbal pain assessment tools…”*

Lack of relevant policies and clinical guidelines was the fourth subcategory obtained from the analysis of “forgotten priority”. The participating ICU nurses highlighted the absence of clinical guidelines on the selection and use of various non-verbal pain assessment tools. Participant #17 stated that:

*“… It is definitely important to me to relieve pain in patients who cannot self-report it... however, the hospital has never introduced a standardized scale to us even though there are various scales in this context to help the personnel to act in the same manner, but not based on their tastes.”*

## Organizational barriers

The participants underscored “organizational barriers” as other challenges faced by ICU nurses. This category contains two subcategories including “inadequate nurse-to-patient ratio” and “presence of less experienced personnel”.

The participants argued that heavy workload and time limitations, consequent to inadequate nurse-to-patient ratio, prevented them from providing constant high-quality care. Participant #12 indicated that:

*“Due to the high workload in the ICU, being responsible for two or more patients admitted into the ICU in each shift, health information system recordings, and paperwork; there is no possibility to use non-verbal pain assessment scales.”*

Analyzing the viewpoints of less experienced nurses (newly employed) showed that their attention and energy was mainly focused on acquiring skills such as working with ICU equipment, doing procedures, and calculating drug dosage. They, hence, had no opportunity to work with non-verbal pain assessment scales. Therefore, the “presence of less experienced personnel” served as another organizational barrier. Participant #9 said that:

*“... My incentives in the ICU are to learn about the mechanical ventilators… I significantly focus on the calculation and regulation of infusion of medicines, the alarms of mechanical ventilators,…”*

## Attitudinal barriers

“Attitudinal barriers” in nurses was another concept derived from data analysis. This category consisted of three subcategories including “adequacy of sedatives”, “failure to understand pain in unconscious patients”, and “no belief in non-verbal pain assessment scales”. Nurses are responsible for pain assessment and should adopt pain-reducing procedures if pain is not relieved. However, the participating nurses believed that there was no need to use pain assessment scales when a patient received sedative infusions. Participant #7 argued that:

*“…there is no need to use pain assessment scales for patients with decreased LOC when drugs such as fentanyl are used in the form of infusion… because they are taking sedatives…”*

Moreover, the subcategory “failure to understand pain in unconscious patients” was extracted from the participants’ statements indicating that patients with decreased LOC could not feel pain. Participant #2 reiterated that:

*“…patients with impaired consciousness have no pain... in fact; they do not feel pain... so it is not necessary to use pain assessment scales for such patients...”*

The participants believed that non-verbal scales could not measure and evaluate pain correctly. They, thus, had “no belief in non-verbal pain assessment scales”. They considered their personal judgments of patient pain as the best pain assessment method. Participant #5 discussed that:

*“... Lots of these pain scales are out of use… they are not 100% correct... I feel that I can evaluate and assess pain… an example is the scale developed for embolism… we had cases in which negative embolism was reported using these scales, but the patient was affected with embolism clinically...”*

## Barriers to knowledge

Another category extracted from data analysis was “barriers to knowledge”. This category contained two sub-categories including “unfamiliarity with the use of non-verbal pain assessment scales” and “insufficient training on the clinical use of pain assessment scales”.

Based on the participants’ statements, undergraduate education did not provide nursing students with adequate knowledge on pain assessment. Therefore, unfamiliarity with pain assessment accounted as a major barrier to pain assessment and measurement. Most participating nurses stated that they had not received adequate training on pain assessment and measurement scales in either school or workplace (hospital). Participant #13 said that:

*“.. well, it is natural that we are kind of familiar with these standardized pain assessment scales... because my colleagues and I, who are working in the ICU, hold undergraduate degrees... well, pain assessment scales are not very often included in the undergraduate programs.”*

Participant #7 highlighted “insufficient training for the clinical use of pain assessment and measurement scales” and argued that:

*“…we have never taken certified training classes in the hospital to become familiar with pain assessment scales as well as the necessity to employ them for patients in the ICU and for those connected to the mechanical ventilator up until now… there have been just sporadic classes in this unit…”*

## Discussion

Four main categories, including “forgotten priority”, “organizational barriers”, “attitudinal barriers”, and “barriers to knowledge” were extracted from the analysis of the experiences of ICU nurses. Specific subcategories of each category were also determined based on unique and integrated properties. This study was among the first Iranian studies to adopt a qualitative approach to explore the experiences of ICU nurses about the use of pain assessment scales. It sought to answer the question: “What challenges are experienced by ICU nurses when using pain assessment tools in patients unable to communicate?”

The findings of this study indicated that although ICU nurses perform routine practices for patients unable to communicate during each shift; they do not follow a routine pain management protocol in this group of patients. Nevertheless, pain management is a major determinant of nursing care quality, i.e. pain should be evaluated when vital signs are measured and its relief should be considered as the core and essence of nursing care [38]. Nurses are also responsible for the prevention or reduction of pain [39]. They are, in fact, one of the important healthcare team members with proper opportunities to assess, identify, and evaluate pain management. They are, hence, required to play an active role in pain management. However, few studies have shown that nurses are actually playing such roles [40].

While nurses’ efforts for pain management mainly aim to improve patient outcomes, there is no appropriate non-verbal pain assessment scale to evaluate pain in ICU patients. It seems that failure in this respect can lead to decreased quality of pain management in patients unable to communicate. According to Bucknall et al. (2007), nurses can only make effective decisions for pain management through the repeated and regular evaluation of pain intensity and related behaviors [41]. Erdek et al. (2004) concluded that there was not an appropriate form of pain assessment in ICU patients and such patients were unable to self-report their pain [42]. A study in Jordan reported that the existing pain assessment methods applied in the ICUs of the country only focused on pain management among patients suffering from cancer. In fact, no particular pain assessment tools were used for ICU patients who are unable to communicate [43]. Similar barriers were reported by ICU nurses in the United States [42].

The experiences of the ICU nurses in this study indicated that physicians’ inattention to pain monitoring, decreased nurse’s attention to pain and its relief. Our participants reported physicians focused on several complications, such as fever, but failed to evaluate pain. Nevertheless, pain relief is an essential human right and a major nursing priority [44].

The absence of non-verbal pain assessment scales in nursing flowcharts is another challenge which ICU nurse’s face while adopting pain management strategies. Currently, the nursing flowchart in these units only uses VAS and SFS to record patient pain. However, there is a need for a standardized form of non-verbal pain assessment and measurement for patients unable to communicate. In the absence of such scales, as well as a specific system for the analysis of their results, the effectiveness of treatments cannot be accurately determined [10]. However, the inclusion of the pain management section in the ICU checklist, as a part of daily activities, can be considered as a valuable scale for reducing patient discomfort [45].

The ICU nurses participating in this study used infusions of sedatives and narcotic drugs for patients unable to communicate without following any pain assessment scales and specific guidelines. Lack of relevant policies and guidelines on pain control was also reported by Keykha et al. (2013) [46]. Nevertheless, lack of access to clinical pain management guidelines can negatively affect pain management [29, 47], i.e. the use of guidelines and non-verbal pain assessment scales would have positive effects on the experience of pain reduction in ICU patients.

Based on the findings of the present study, the undesirable nurse-to-patient ratio in the ICUs and nurses’ heavy workload forced nurses to disregard some clinical practices and prevented them from the frequent use of pain assessment tools. The time limits could also interfere with the quality of care and were thus considered as a barrier to optimal care [48]. On the other hand, limited time forced nurses to prioritize duties of equal importance [49]. Unfortunately, the alarming shortage of nurses is considered as an important challenge in healthcare systems [50, 51]. In Iran, there is a need for over100 thousand more nurses [52].

Apart from the issue of time, experiences and skills of the nurses are similarly critical in pain diagnosis [53]. The less experienced ICU nurses recruited in this study had no opportunities for performing pain measurement and working with non-verbal pain assessment tools because they were mostly interested in the acquisition of other skills (e.g. working with the MV and other equipment).

The findings of this study highlighted the viewpoints of ICU personnel’s as other factors influencing the use of pain assessment scales. In fact, pain management often depends on the viewpoints, culture, and beliefs of the health-care team [54]. The ICU nurses in this study believed that there was no need to use pain assessment scales for patients receiving sedatives. Examining their viewpoints and experiences also revealed that the personnel did not feel any need to assess pain in patients when they were receiving pain-killers and sedatives prior to performing invasive and painful procedures. The findings of a study in this respect also showed that most patients under an MV received sedatives and pain-killers without any particular pain assessment [35]. However, prescribing the correct dosage of sedatives in patients with decreased LOC requires the routine administration of pain assessment tools [55]. Enskar et al. (2007) showed that Swedish nurses had more knowledge about pain assessment and more positive attitudes towards pain. These factors could lead to better pain relief [56].

“Failure to understand pain in unconscious patients” was another concept derived from the experiences of the ICU personnel in this study. The nurses argued that patients with decreased LOC had no pains, i.e. pain assessment and scales were not necessary for these patients. Likewise, nurses in other investigations mainly neglected pain in unconscious patients. They did not actually consider pain as a serious issue since they assumed that patients with decreased LOC did not have a sense of pain [57]. However, the point of importance is that the state of sleep and sedation is not equal to the absence of pain or its relief [14]. It is difficult to evaluate pain in such patients due to the inability to communicate following decreased LOC, receiving sedatives, and using the MV. Consequently, inadequate pain management and control in unconscious patients has been raised as a challenge in nursing care [58].

The final concept obtained from this category of experiences by ICU nurses was “no belief in pain management scales”. The nurses did not believe in pain scales and argued that personal judgment of the patient’s pain was the best method of pain assessment because they had experiences of ineffective use of other tools such as the scale for embolism. Given their high workload and time limits, these nurses also believed that they could assess patients’ pain only through the patient’s face and observation of their hemodynamics. Other studies have also mentioned personal beliefs and viewpoints as major barriers in this respect. The personnel’s lack of belief can thus lead to treating patients based on their personal opinions [59]. Given that nurses need tools to correctly assess pain [39, 60], they should avoid personal assessment and judgment in this respect.

The concept of “barriers to knowledge” indicates that “unfamiliarity with non-verbal pain assessment scales” and “inadequate ability to use non-verbal pain assessment scales” are among the main challenges in this domain. In the present study, the ICU nurses did not use pain measurement scales because they received little information in their undergraduate programs or in-service re-training courses about pain assessment scales. Most nurses believed that they were not well prepared for this function during their training courses presented in nursing education centers [61].

Moreover, Rose et al. (2012) examined the performance of ICU nurses regarding pain management and control. They reported that nurses were not willing to use pain assessment scales in non-verbal patients and that they had little information about such scales, which could negatively affect their performance in terms of patient pain management [62].

In this regard, Farahani et al. (2008) stated that inadequacy of training courses for pain measurement was one of the significant barriers to its use [63]. Therefore, training pain assessment scales, their use and the related guidelines are of utmost importance for improving systematic pain assessment in ICU patients and ultimately for increasing nurses’ knowledge of pain care.

## Conclusion

The findings of the present study indicate that various factors such as “forgotten priority”, “organizational barriers”, “attitudinal barriers”, and “barriers to knowledge” could affect the use of scales for pain assessment and management in patients unable to communicate. Given the inability to self-report in these patients, pain cannot be properly assessed and treated in such patients. The existing barriers to using non-verbal pain assessment scales in these patients can also lead to false evaluations of pain by nurses and consequently unrealistic perception of pain and inadequate medication. Identifying these challenges for nurses can help take effective steps such as empowering nurses in the use of non-verbal pain assessment scales, relieving pain, and improving the quality of care services.

## Abbreviations

ICU: Intensive care unit
LOC: Decreased level of consciousness
MV: Mechanical ventilator
